# Resting State EEG Hemispheric Power Asymmetry in Children with Dyslexia

**DOI:** 10.3389/fped.2016.00011

**Published:** 2016-02-24

**Authors:** Eleni A. Papagiannopoulou, Jim Lagopoulos

**Affiliations:** ^1^Brain and Mind Research Institute, University of Sydney, Camperdown, NSW, Australia; ^2^Sunshine Coast Mind and Neuroscience – Thompson Institute, University of the Sunshine Coast, Birtinya, QLD, Australia

**Keywords:** dyslexia, children, hemispheric lateralization, auditory processing, electroencephalography, EEG

## Abstract

Dyslexia is a neurodevelopmental disorder estimated to affect between 4 and 7% of the population. It is often referred to as a learning disability and is characterized by deficits in the linguistic system. To better understand the neural underpinnings of dyslexia, we examined the electroencephalography (EEG) power spectra between pre-adolescents with dyslexia and neurotypical control children during eyes closed state. We reported the differences in spontaneous oscillatory activity of each major EEG band (delta, theta, alpha, and beta) adopting a global as well as in a region-by-region and hemispheric approach to elucidate whether there are changes in asymmetry in children with dyslexia compared to controls. We also examined the relationship between EEG power spectra and clinical variables. The findings of our study confirm the presence of an atypical linguistic network, evident in children with dyslexia. This abnormal network hallmarked by a dominance of theta activity suggests that these abnormalities are present prior to these children learning to read, thus implicating delayed maturation and abnormal hypoarousal mechanisms.

## Introduction

Dyslexia is a neurodevelopmental disorder of unknown etiology estimated to affect between 4 and 7% of the population (1). The disorder is often referred to as a learning disability and is characterized by core cognitive deficits in the linguistic system, specific to phonological processing (2). Additional deficits include functions specific to the magnocellular visual pathways (3–5), noise exclusion (6), sluggish attention shifting (7), rhythmic entrainment (8, 9), and abnormal cerebellar function, associated with dystonia and dyscoordination (10).

Early studies in dyslexia have highlighted abnormalities related to hemispheric lateralization, postulating that children with dyslexia fail to exhibit the expected left-hemispheric specialization that allows visual representations and phonological integration of printed information Orton (11, 12). Hemispheric lateralization effects in dyslexia have been examined in structural post-mortem studies (13, 14) as well as functional imaging studies (15–17) during cognitive processing conditions and have been identified in areas implicating encoding, speech perception, and transferring of information between the two hemispheres. Collectively, early studies have yielded distinct activation patterns between impaired and non-impaired readers, reporting hypoactivation of brain regions of the (i) left-hemispheric regions, including inferior frontal, superior temporal, parieto-temporal, and middle-temporal, middle-occipital gyri and (ii) right-hemispheric regions that include the inferior frontal, superior temporal, cingulate, and medial orbital gyri. The aforementioned findings have been corroborated by a recent study, whereby children with dyslexia failed to exhibit left-hemispheric dominance during a phonological working memory condition (18).

Non-invasive measures of brain function such as those from electroencephalography (EEG) studies (examining mostly pre-adolescent children with dyslexia) have provided additional insights into cortical lateralization models. The predominant findings that have emerged from these studies have been the attenuation in the power of faster frequencies (such as alpha and gamma) and an increase in the power of slower frequencies (such as delta and theta). These findings would suggest that in addition to the lateralization processes, children with dyslexia may also be experiencing maturational lag and possibly cortical hypoarousal, as reflected by predominance of the slower frequencies. Attenuation of high beta power at central and bilateral mid-temporal regions in children with dyslexia has been reported by Fein et al. (19). Earlier to this, Duffy et al. (20) had reported an increase in alpha power in left-hemispheric temporal areas, as well as in the left parietal and left posterior–central and frontal areas in dyslexia, corroborating previous findings of a relative increase of low frequency power in left-hemispheric parieto-occipital areas; where children with dyslexia had increased delta/theta (3–7 Hz) power, compared to neurotypically developing controls who had increased alpha power (9–14 Hz) (21).

Using intra-cortical power spectra EEG at both resting state and syllable processing, Morillon et al. (22) detected a hierarchical interaction between right-hemispheric high-theta (7–8 Hz) and left-hemispheric low gamma (25–45 Hz) frequency bands, which was proposed to underpin the mechanism of neural coding shifts from stimulus to phonological encoding. Specifically at rest, significant inter-hemispheric asymmetry was noted in the theta band in both spectral and spatial domains; left-hemispheric regions reached higher power dominance (~7.5 Hz), compared to right-hemispheric regions (~5.5 Hz). The results of this study suggests that left-hemispheric regions preferentially sample and integrate acoustic information from short temporal windows ~20–40 ms, at gamma rate (~25–45 Hz), whereas right-hemispheric regions preferentially sample and integrate information from longer ~150–250 ms temporal windows, at a theta rate (5–6 Hz). These findings corroborate the “asymmetric sampling in time” hypothesis (23), where it has been posited that the speech signal is neutrally represented in a bilateral and symmetrical fashion at an early stage but is further elaborated asymmetrically in the time domain. This hypothesis could explain functions such as parsing speech signals into segments, allowing for vowel identification and subsequent integration of phonemic and syllabic information, at a gamma and theta rate, respectively.

Spontaneous oscillatory activity at resting state is a hallmark of internal models and states (24), and reflects experience and developmental state (25). It hallmarks resting neuronal network status across cortical regions and is implicated in the representation of information, regulation of information flow as well as its storage, and recall (26–30). Notably, it is thought to reflect baseline perceptual and cognitive processing and, thus, probing the resting state of these networks provides a reliable representation of brain homeostasis. This is critical in as much as it allows fundamental neurobiological characteristics specific to dyslexia to be identified, and provides the ability to elucidate how impairments in the EEG might contribute to downstream network dysfunctions. Eyes closed resting state EEG also has the potential to provide insights into distinct patterns of oscillatory activity, before task-relevant cortical activation takes place.

In the present study, we sought to examine the differences in EEG power between pre-adolescents with dyslexia and neurotypical control children. To our knowledge, no EEG study in the last decade has investigated hemispheric power asymmetry in these children with dyslexia at a resting (eyes closed) state. In this study, we report the differences in spontaneous oscillatory activity of each major EEG band (delta, theta, alpha, and beta) adopting a global as well as in a region-by-region and hemispheric approach to elucidate whether there are baseline changes in asymmetry in children with dyslexia compared to controls. We also examine the relationship between EEG power spectra and clinical variables. The findings that emerge from this study allow us to reliably interpret results at a fundamental level; prior to task performance and, thus, explore the relevance of maturational lag and hypoarousal models in children with dyslexia. At a primary, cognitive state, we hypothesized that compared to controls; children with dyslexia will show attenuated left-hemispheric specialization as measured by EEG power spectra.

## Materials and Methods

### Participants

Twenty-one participants with dyslexia (mean age = 8 years 4 months, SD = 1.40, 9 males) and 19 normal readers (mean age = 8 years, 2 months, SD = 1.64, 11 males) were recruited into the study. The participants with dyslexia were recruited via the Australian Tutoring Association, and the Specific Learning Difficulties Association, New South Wales. The control subjects were recruited separately to the dyslexia cohort via advertisements placed in participating schools as well as snowball sampling techniques. All participants reported normal hearing and normal or corrected to normal vision. No history of ADHD, neurological disorders or brain injury was reported. All participants were required to have adequate intelligence as defined by an IQ score of >85 on the Wechsler Abbreviated Scale of Intelligence, Second Edition (WASI-II). Dyslexic and non-dyslexic participants were matched based on (1) age and (2) IQ.

The study was conducted at the Brain and Mind Research Institute of the University of Sydney. Informed written consent was obtained from parents or caregivers along with verbal assent from the participating children, in accordance with the Declaration of Helsinki. This study was approved by the University of Sydney Ethics Committee.

### Behavioral Assessments

All participants were administered the two subtests of the WASI-II; a verbal ability subtest (Vocabulary) and a visual-spatial ability subtest (Matrix Reasoning). The Dyslexia Early Screening Test – Second Edition (DEST: children up to 6 years, 5 months) and Dyslexia Screening Test – Junior (DST-J: children 6 years and 6 months to 11 years and 5 months), research valid and reliable instruments (31) assessed children’s performance with respect to dyslexia based on the following criteria of the two instruments used. Two children aged <6 years and 5 months (DEST) were assessed on: (1) Rapid Naming, (2) Bead Threading, (3) Phonological Discrimination, (4) Postural Stability, (5) Rhyme/First Letter, (6) Forward Digit Span, (7) Digit Naming, (8) Letter Naming, (9) Sound Order, (10) Shape Copying, (11) Visual spatial memory, and (12) Vocabulary. Nineteen children aged 6 years and 6 months, to 11 years and 5 months were assessed on: (1) Rapid Naming, (2) Bead Threading, (3) One-Minute Reading, (4) Postural Stability, (5) Phonemic Segmentation, (6) Two-Minute Spelling, (7) Backwards Digit Span, (8) Non-sense Passage Reading, (9) One-Minute Writing, (10) Verbal Fluency, (11) Semantic Fluency, and (12) Vocabulary. The following tests overlapped across both the DEST and the DST-J in terms of the psychological domains which they probed and as such all scores were used to create the overall average for Rapid Naming, Phonological Segmentation, One-minute reading, Non-sense Passage, Postural Stability, and Vocabulary. Children with an At-Risk Quotient (ARQ) of >0.6 formed the dyslexia group and children with an ARQ < 0.4 formed the control group. No participant with dyslexia had an ARQ < 0.6 and similarly no control participant had an ARQ > 0.4. Participant details and group scores are presented in Table 1.

**Table 1:** **Linguistic and cognitive measures**.

Measures	Controls (*n* = 19)		Dyslexic (*n* = 21)		*t*	*p*
	M	SD	M	SD		
Age	8.2	1.64	8.4	1.40	0.62	>0.05
Full scale IQ-2 (WASI-II)	109.26	10.6	101.05	9.1	1.2	>0.05
At-risk quotient	0.22	0.12	0.92	0.26		<0.005
Rapid naming	37.8 s	9.33	56.2	24.50	3.07	<0.004
Phonological segmentation	11.2	1.03	8.1	2.05	5.86	<0.005
One-minute reading	69.6	17.75	32.9	21.47	5.86	<0.005
Non-sense passage reading	56.2	11.04	39	15.89	3.91	<0.005
Postural stability	2.5	3.25	7.7	4.14	4.37	<0.005
Vocabulary	13.4	1.34	12.4	2.29	1.73	>0.005

### EEG Procedure

Electroencephalography was recorded for 3 min in a resting eyes closed condition, using a Compumedics Quik-Cap from 19 electrode sites (Fp1, Fp2, Fz, F3, F4, F7, F8, Cz, C3, C4, T7, T8, Pz, P3, P4, P7, P8, O1, and O2) using the International 10–20 System. Linked mastoids served as reference. Eye movement activity was monitored via electooculography (EOG) using two bipolar electrodes placed 1 cm lateral to the outer canthus of each eye to measure horizontal EOG, and a separate bipolar electrode set was placed above and below the center of the left eye to record vertical eye movements. All subjects were seated in a comfortable chair and electrode impedances were maintained <5 kOhms. All potentials were recorded on a Neuroscan Synamps2 DC system (Compumedics, Abbotsford, VIC, Australia) using a sample rate of 1000 Hz, notch filter of 50 Hz and bandpass filter 0.05–100 Hz. All EEG recording was continuous and EOG correction was carried out post acquisition using the technique of Gratton et al. (32) in which linear regressions were calculated between each of the EOG and the EEG channels. Regression coefficients were then determined, from which correction factors were derived and applied to correct the EEG data.

### Data Analysis

All data analyses were undertaken using the Analyzer2 software package (33). Fast Fourier transform (FFT) analysis of 180 artifact-free 1-s epochs was used to determine absolute EEG activity (power) in the delta (0.5–3.5 Hz), theta (3.6–7.4 Hz), alpha 1 (7.5–10.5 Hz), alpha 2 (10.6–12.4 Hz), and beta (12.5–30.0 Hz) bands.

Our objective was to compare the relative power in resting EEG of children with dyslexia to those of controls for the following analyses: (a) across all frequency bands, (b) frontal vs. central vs. posterior-occipital regions (c) Broca’s Area and Wernicke’s Area, and finally (d) left vs. right hemispheres. Age and gender were both used as covariates in the EEG analyses.

Each frequency band analysis was submitted separately to a repeated-measures’ two-way ANOVA, in which group (Dyslexia vs. Controls) was a between-subject factor and site was a repeated within-subject factor. Between- and within-group comparisons were undertaken. Initially a between-group analysis was undertaken across all sites. This was followed by a between-group regional analysis that examined frontal (Fp1, Fp2, Fz, F3, F4, F7, F8), central (Cz, C3, C4), and posterior-occipital (Pz, P3, P4, P7, P8, O1, O2), Broca’s Area (F7, F3, C3), Wernicke’s Area (T7, P7, P3), total left-hemispheric (Fp1, F3, F7, C3, T7, P3, P7, O1), and right-hemispheric (Fp2, F4, F8, C4, T8, P4, P8, O2) activity. To obtain normality of distribution and homogeneity of variance, absolute power scores were log transformed and relative power scores (*x* %) calculated using Log[*x*/(100 − *x*)] (34).

With repeated-measures design, Greenhouse–Geyser correction was used for adjusting univariate results for violations of compound symmetry assumptions. Missing values were replaced by predicted values using regressions on the surrounding sites of the same power band for the control and dyslexia groups separately. Bonferroni-type adjustments were applied to control for type I error.

## Results

### Clinical Variables

There were no significant differences in mean age (*t* = 0.62; *p* < 0.8) between children with dyslexia and controls. Mean WASI-II FSIQ2 score for dyslexia children was 101.05 (SD = 9.1) and controls 109.26 (SD = 10.6). There was no significant difference between groups in WASI-II scores (*t* = 1.2; *p* < 0.1). Dyslexia children have significantly higher mean rating scores on the DST [dyslexia mean = 0.92 (0.26); controls mean = 0.22 (0.12); *p* < 0.005]. Mean DST-J (*n* = 19) and DEST-II (*n* = 2) score for dyslexia children was 0.9 (SD = 0.26) and controls 0.2 (SD = 0.12), *p* < 0.005. Differences between the two groups were identified in rapid naming (*p* < 0.004, *t* = 3.07), phonological segmentation (*p* < 0.005, *t* = 5.86), one-minute reading (*p* < 0.005, *t* = 5.86), non-sense passage reading (*p* < 0.005, *t* = 3.91), and postural stability (*p* < 0.005, *t* = 4.37).

### EEG within Group Results

#### Region Analysis

Children with dyslexia did not exhibit any significant EEG power changes across frontal, central, and parietal regions for any EEG frequency band. Instead, control subjects exhibited significantly decreased frontal (mean = 0.72; SD = 3.6; range = 1.2) Theta EEG power, when this was compared to central (mean = 1.65; SD = 1.4; range = 5.6) and parietal (mean = 1.57; SD = 1.3; range = 4.7) regions [*F*(5.12); df(2,17); *p* = 0.018].

#### Language Analysis

The results for the Broca’s and Wernicke’s area analysis revealed that children with dyslexia had significantly greater Delta [*F*(25.97); df(1,20); *p* < 0.005] and Theta [*F*(4.59); df(1,20); *p* = 0.045] EEG power at Broca’s (mean = 4.38; SD = 2.3; range = 9.6) when compared to Wernicke’s area (mean = 1.02; SD = 1.6; range = 5.3). The control children did not exhibit any EEG band power asymmetry in either area, for any of the EEG bands.

#### Hemispheric Analysis

Children with dyslexia had significantly decreased EEG power in the left (mean = 0.12; SD = 0.2; range = 0.5), compared to the right hemisphere (mean = 0.15; SD = 0.2; range = 0.8) for Alpha2 [*F*(5.97); df(1,20); *p* = 0.024] and Beta [*F*(4.957); df(1,20); *p* = 0.038] frequency bands, whereas control subjects had significantly decreased EEG power in the left hemisphere (mean = 0.74; SD = 0.4; range = 1.8) for the Theta band [*F*(5.625); df(1,18); *p* = 0.029].

### EEG between Group Results

**Figure 1:**
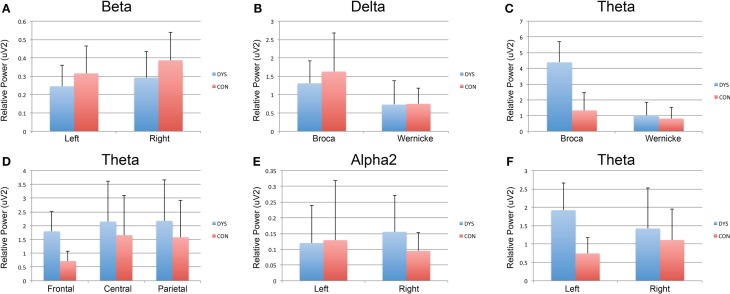
**Within-group analysis of EEG band for region, language, and hemisphere**. Dyslexia subjects are depicted in blue and controls in red. **(A)** Significantly decreased EEG power in the left compared to the right hemisphere for Beta in children with dyslexia. **(B,C)** Significantly greater Delta and Theta EEG power, respectively, at Broca’s when compared to Wernicke’s area. **(D)** Significantly, decreased frontal theta EEG power when compared to central and parietal regions for control subjects. **(E)** Significantly decreased EEG power in the left compared to the right hemisphere for Alpha2 EEG power in children with dyslexia. **(F)** Significantly decreased EEG power in the left compared to right hemisphere for the Theta EEG power in controls.

**Figure 2:**
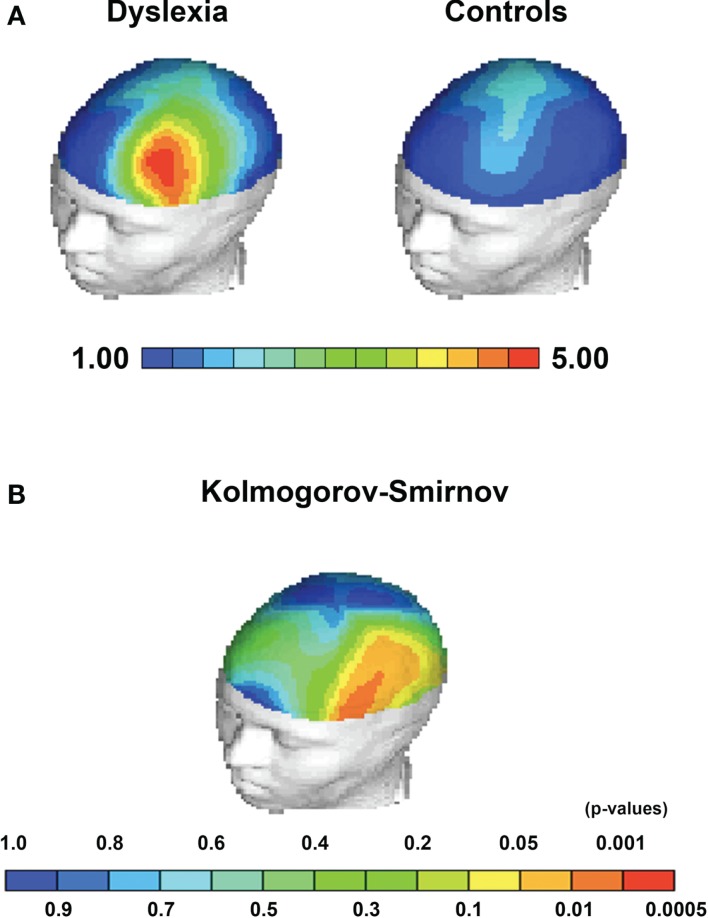
**Statistical topographical maps depicting group average differences and KS-statistics for Theta EEG power**. **(A)** Group average Theta power differences between children with dyslexia and controls localized to Broca’s area. **(B)** Kolmogorov–Smirnov between group statistic indicating significant topographical between group differences in Broca’s area, frontal regions, and the left hemisphere.

Children with dyslexia had significantly greater Theta power in the frontal region [*F*(5.25); df(1,38); *p* = 0.028] (mean = 1.79; SD = 2.0; range = 7.7), also significantly greater Theta power in Broca’s Area [*F*(6.34); df(1,38); *p* = 0.016] (mean = 4.38; SD = 2.3; range = 9.6) and greater Theta power in the left hemisphere [*F*(4.74); df(1,38); *p* = 0.36] (mean = 1.9; SD = 3.4; range = 8.3), when compared to controls (see Figures 1 and 2).

## Discussion

The overarching aim of the present study was to examine group differences in spontaneous oscillatory brain activity during a resting (eyes closed) condition. EEG power was examined across all frequency bands in children with dyslexia and contrasted to neurotypical children. Spectra analyses were examined across (i) frontal, central, and posterior brain regions, (ii) Broca’s and Wernicke’s area, and finally, (iii) left and right hemispheres. The results of these analyses revealed significantly increased theta power for the dyslexia group (when compared to controls) in frontal brain regions, the scalp topography corresponding to Broca’s area and greater theta power in the left hemisphere. Children with dyslexia also had significantly increased slow wave activity (for both delta and theta), in Broca’s compared to Wernicke’s area, which was in direct contrast to the control children who did not exhibit any asymmetry across these two areas. Children with dyslexia also had significantly decreased EEG power on the left for alpha2 and beta frequency bands, but had significantly increased EEG power in the left hemisphere for the theta band. Decreased resting state beta and alpha power is in agreement with previous findings by Fein et al. (19), although the authors observed this trend bilaterally at central and mid-temporal areas in children with dyslexia. The decreased alpha power was not observed by Duffy et al. (20) who, on the contrary, reported an increase in alpha power in the left-hemispheric temporal areas, as well as in the left parietal and left posterior–central as well as frontal areas in children with dyslexia during resting state. However, direct comparison of these findings to our results is limited due to significant differences in the way the statistics were conducted. For example, the Duffy et al. (20) study did not formally test differences between the control and dyslexia groups. Instead, they reported group differences were based on a percentile index which as the authors stated was “…not used to measure the overall statistical significance of group separation….”

Evidence suggests that theta band power underlies the mechanism by which cognitive control is realized (35) and more recently it has been shown that increased phonological activity results in increased activations in the frontal brain regions, the mechanism of which has been hypothesized to involve cognitive control (36). Thus, on the basis of this evidence, the increased theta power in frontal and Broca’s regions from our study may be indexing similar processes to previous studies, which have reported increased frontal lobe and Broca’s area activation during phonological tasks (37, 38). These are thought to underpin the core neurophysiologic correlates of linguistic computation, implicated in word-level processing (i.e., assembling phonological information), semantic, and syntactic processing. Similar abnormalities with respect to Broca’s area in dyslexia subjects have also been reported by PET studies during auditory phonological/rhyming tasks (39), and in regard to lateralization, our theta findings are consistent with those first reported by Sklar et al. (21). However, the authors of this study observed this result over the parieto-occipital region, rather than frontal areas as was the case in our study. Collectively, our findings confirm that at a resting state, the EEG of children with dyslexia significantly differ from matched controls in theta frequency across distributed cortical locations (i.e. frontal and Broca’s area as well as the left hemisphere) which are integral to the processing of language-related information.

Structural studies in dyslexia date back to the seminal work of Broca and Wernicke (40, 41), and more recently those of language production as well as specific aspects of syntactic processing (42, 43), which are primarily localized in the left hemisphere. These studies have unambiguously described how language-related regions show profound left hemisphere lateralization [for a review, see Toga and Thompson (44)]. This work has precipitated further investigation of these language-related areas in children with dyslexia and the results for the most part support the notion that these regions are adversely impacted in individuals with dyslexia.

More recently, studies employing functional imaging paradigms have attempted to characterize the workings of the so-called “dyslexic reading network” in children (18, 45) with dyslexia, and have reported frontal brain region abnormalities. Specifically, they have highlighted functional abnormalities in the left inferior frontal gyrus during phonological and rapid naming tasks.

The significant increase of theta power in our dyslexia group provides strong support for the presence of downstream event-related band power changes during task demands. Such changes have been defined as “tonic” and “phasic,” reflecting decreased (resting) and increased (active) performance, respectively. Both are volitionally controlled and mostly occur at a rapid rate. In this regard, it has been shown that theta power synchronizes (phasic change) with increasingly task demands (46, 47). During increased task performance, theta power is observed to increase and as such increased phasic theta power in response to task demands has been shown during successful encoding of new information (48). Notably, in our study the control group had low theta activity as would be expected during resting conditions. However, the children with dyslexia exhibited a significant increase in tonic (i.e., in the absence of cognitive/stimulus load) theta and these observations were localized to the left frontal hemispheric regions. This finding highlights the presence of specific resting state (tonic) functional abnormalities in dyslexia and may indicate aberrant activation/desynchronization formed prior to children’s learning to read. Lateralized patterns of aberrant theta activation, which include delayed peak theta activity (49) as well as sustained theta EEG peak activity (50), have also been reported during phonological processing tasks (in adults). The exact origins of these abnormalities are not known; however, work conducted in animals examining the mechanisms that modulate theta activity shows how theta is reliably increased following GABAergic inhibition (51). Thus, the observed increase in low frequency activity during eyes closed in children with dyslexia is a strong indicator of the presence of atypical network activity and, collectively, the aforementioned studies suggest that these theta abnormalities may represent a putative neurobiological marker, reflecting CNS disinhibition. This notion is supported by other neurophysiologic studies that have reported decreased P3 event-related component in dyslexia (52–54), which is a positive-going component occurring 250–500 ms following a low frequency stimulus, otherwise embedded in a train of more frequent occurring stimuli. The P3 is conjectured to reflect context updating mechanisms (55) as well as the inhibitory state of the cortex. In this regard, magnocellular and intralaminar mechanisms have been posited to regulate cortical excitability (56, 57) via the polarization of apical dendrites, which are known to contribute to the EEG. Notably, previous studies have reported network dysfunctions within magnocellular and intralaminar networks in dyslexia and a recent magnetic resonance spectroscopy study has also reported increased glutamate in people with reading disabilities.

Central nervous system homeostasis is essential for a stable resting state and efficient cognitive processing. Homeostasis is achieved via a delicate interplay between excitatory and inhibitory neurotransmitters that act on different receptors, which differentially produce effects on the excitability of groups of neurons. Reflecting this need for a highly integrated system of excitatory and inhibitory control of neuronal activity, the metabolic pathways regulating the brain’s main excitatory and inhibitory neurotransmitters are tightly coupled and any dysregulation of these systems can result in an imbalance in cortical excitability and, thus, CNS instability. Collectively, the aforementioned studies lend support to the notion that the mechanisms that underpin dyslexia may be modulated by changes in cortical excitability and in particular disinhibition.

## Conclusion

The findings of our study confirm the presence of an atypical linguistic network, evident at a resting state in children with dyslexia. This network is hallmarked by a dominance of theta activity in the left frontal regions and as such, future studies should explore the relationship between phasic theta (as acquired in an activation task), downstream electrophysiological indices such as the P300 event-related potential with behavioral measures of dyslexia.

## Author Contributions

All authors listed have made substantial, direct and intellectual contribution to the work, and approved it for publication.

## Conflict of Interest Statement

The authors declare that the research was conducted in the absence of any commercial or financial relationships that could be construed as a potential conflict of interest.
